# Aggressive fibromatosis in pediatric population—A case series

**DOI:** 10.1016/j.ijscr.2018.09.032

**Published:** 2018-10-04

**Authors:** Masood Umer, Javeria Saeed, Nida Zahid

**Affiliations:** Department of Surgery, Aga Khan University Hospital, Stadium Road, P. O. Box 3500 Karachi 74800, Pakistan

**Keywords:** Aggressive fibromatosis, Pediatric patients, Disease free survival, Positive margins

## Abstract

**Introduction:**

Aggressive fibromatosis or Desmoid fibromatosis is a benign tumor which originated form mesenchymal tissues of the body. This tumor has strong potential of recurrence and infiltration but it does not metastasize to other organs of the body. This case series is focused to determine the treatment outcomes for pediatric patients of aggressive fibromatosis.

**Methodology:**

It is a retrospective case series conducted on 7 patients presented to section of Orthopedics, department of surgery of our institute in Karachi. We included all the cases of pediatric patients from 1 to 16 years, with biopsy proven fibromatosis from January 2000 to December 2015.

**Presentation of Cases:**

Out of 7 pediatric patients, there were 6 (85.7%) males and 1 (14.3%) female patient. The median age was 6 years IQR (5–11) years. Gluteal region was the most common site of disease. Four patients (57.1%) had positive tumor margins while three (42.9%) had negative margins. Out of 7 patients, 4 patients (57.1%) had recurrent disease and they had positive margins. The median Disease Free survival time was 14 months and there was no expiry of patients.

**Conclusion & recommendation:**

The conclusion of our study was that aggressive fibromatosis is more prevalent in children below 15 years of age and disease burden is higher in male gender. Positive margins after surgery indicate a high risk for disease recurrence therefore; primary surgery with negative margins is the treatment of choice for children with AF. However, we recommend that multicenter trials should be conducted in the future to clarify the role of adjuvant treatment for patients with pediatric AF.

## Highlights

• Desmoid tumor or aggressive fibromatosis (AF) is a benign tumor of borderline malignant condition, it is infiltrative, deep-seated and muscoloapo neurotic in nature.
• AF is commonly found among children below 15 years of age, affecting males more as compared to females.
• Primary surgery is not the treatment choice among patients with positive margins as the risk of recurrence is higher after surgery. However, radiation and chemotherapy may assist in maintaining local control of these tumors in such patients.
• Primary surgery with negative margins is the treatment choice for children with AF.

## 1 Introduction

Desmoid tumor or aggressive fibromatosis (AF) is a benign tumor of borderline malignant condition, it is infiltrative, deep-seated and muscoloapo neurotic in nature. World health organization (WHO) defines fibromatosis as a tumor that originates from mesenchymal tissues which is non-metastatic but presented as locally belligerent lesion which accounts for 0.03% of neoplasms and 3% of every type of soft tissue lesion. Although it is non-metastasizing tumor but it has high frequency of recurrence [1]. The incidence rate of AF per year is 0.2–0.4 per 100,000 populations and there are two peaks of incidence among children falling in age group of 6 years – 15 years and from age of puberty to 40 years of age in females. It is a locally destructive tumor that arises from connective tissue, muscoloaponeurotic and fascial muscle sheath that undergoes fibroblastic proliferation. AF has a very high predisposition for local recurrence [2,3].

In children, the mean age for diagnosis of AF is 8 years with age range 0–19 years and majority are boys. However, it is observed that the incidence of AF is much higher in children with positive family history of aggressive fibromatosis, Gardner’s syndrome and adenomatous polyposis. Children present with slow growing, painless mass. Although the pathogenesis of these tumors is ambiguous but an important factor that may be responsible for its occurrence could be the deregulation of beta-catenin pathway in which the tumor suppressor gene is responsible for maintaining the beta-catenin levels which in turn changes the nuclear signaling and translation of its pathway [4].

Records of 7 patients were retrieved from January 2000 to December 2015, presented to section of Orthopedics, department of surgery of our institute in Karachi. We included all the pediatric patients ranging 1–6 years, with biopsy proven fibromatosis. Patients of other border line benign /malignant conditions were excluded. Since the data of the patients was retrieved via records therefore Ethical exemption was taken from the ethics review committee of our institute. The aim of this study is to analyze the long term outcomes of seven cases of pediatric fibromatosis presenting to a tertiary care hospital in Karachi, Pakistan.

## 2 Case series

**Fig. 1:**
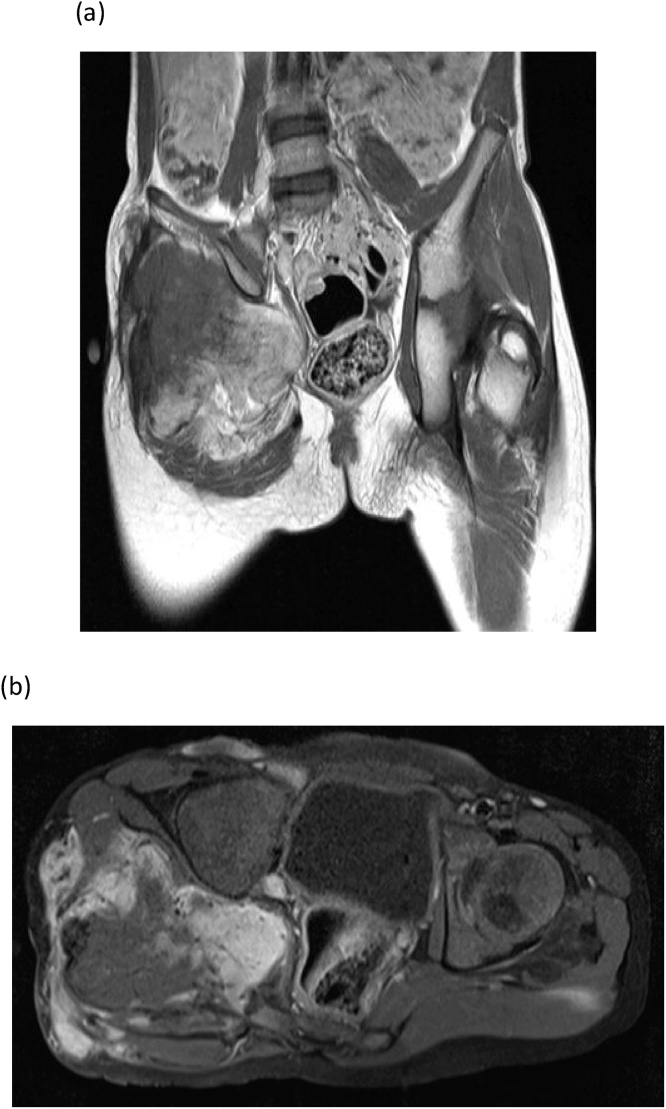
MRI pelvis of fibromatosis of right gluteal region. Coronal plane showing mass infiltrating in the adjacent gluteus muscles and entering in pelvis. Axial plane showing no lymphadenopathy and normal vessels.

Out of 7 pediatric patients, there were 6 (85.7%) males and 1 (14.3%) female patient. The median age was 6 years IQR (1–16) years. The most common site of AF was gluteal region, 4 (57.1%), followed by distal forearm 1(14.3%), axilla 1 (14.3%), knee 1 (14.3%). All of the patients had a common clinical presentation of swelling in the lesion affected site for at least 3 months and only 2 patients had pain symptoms. Patients were advised MRI and X –ray in radiological imaging. The MRI pelvis of one of the patient diagnosed with fibromatosis of right gluteal region showed the mass lesion infiltrates into the adjacent gluteus muscles and enter into the pelvis through the sciatic foramen. It is indenting the urinary bladder and rectum, however the fat planes appear to be preserved. No significant pelvic lymphadenopathy noted and the visualized vessels showed normal flow voids (Fig. 1a & b).

**Fig. 2:**
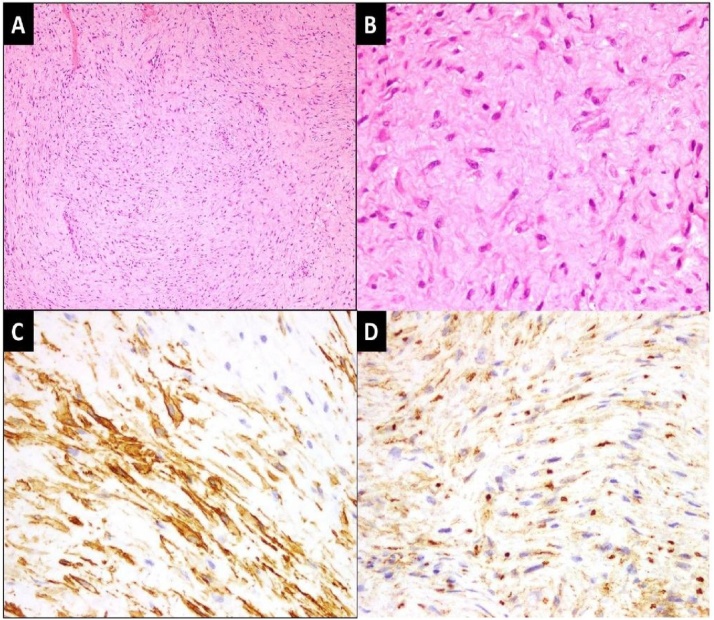
Pre-operative (incisional) biopsy. [A] Low power view of the lesion exhibiting fascicles of spindle cells against fibrotic background. (H&E stain; 100x magnification) [B] High power view of spindle cells with bland, elongated and vesicular nuclei. (H&E stain; 400× magnification) [C] Spindle cells showing patchy expression for alpha smooth muscle actin and [D] patchy nuclear expression for β-Catenin IHC stains. (400× magnification).

**Fig. 3:**
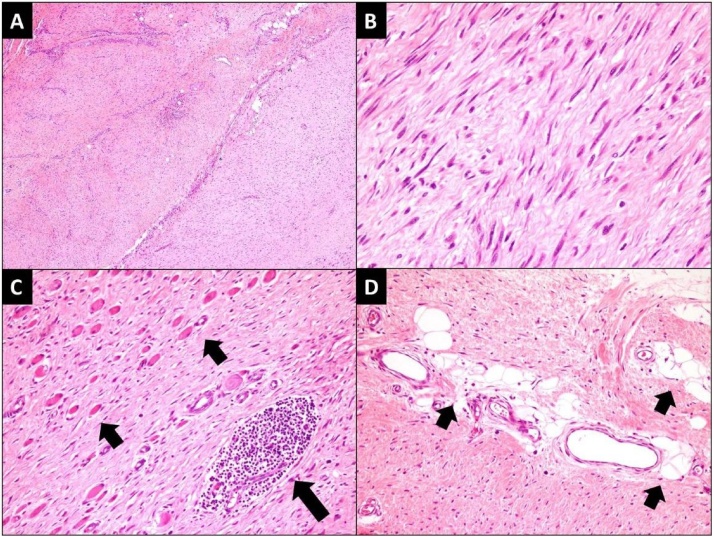
Post-operative (excisional) biopsy. [A] Low power view of the lesion exhibiting fascicles of spindle cells against fibrotic background. (H&E stain; 100× magnification) [B] High power view of spindle cells with bland, elongated and vesicular nuclei. (H&E stain; 400× magnification) [C] Entrapped atrophic skeletal muscle fibers (short arrows) and lymphoid aggregate (large arrow). (H&E stain; 200× magnification) [D] Entrapped adipose tissue (lshort arrow). (H&E stain; 200× magnification).

4(57.1%) of the children had positive tumor margins while 3 (42.9%) had negative tumor margins. Recurrence of disease occurred in 4 (57.1%) patients, while 3 (42.9%) had no recurrence (Table 1). Out of the 4 patients that had recurrent disease, 3 patients had recurrence at 28 months, 34 months and 14 months respectively while one patient had 3 recurrences at 23 months, 35 months and 71 months. The median Disease Free survival time was 14 months. All patients were alive on last follow up, completing 15 years of survival; only one patient was lost to follow up. Moreover, 6 (85.7%) patients received no adjuvant therapy. Only 1 patient who had multiple recurrence had, received radiation therapy on the third recurrence in other country. Other patients with recurrence did not receive any adjuvant therapy because in our country, the radiation oncologists are not having unanimous opinion on this. Only negative surgical margins are main treatment plan. All the patients underwent wide margin excision after preoperative incisional biopsy for diagnosis. Figs. 2 & 3 shows pre-operative (incisional) biopsy and post-operative excisional biopsy respectively. The research work has been reported in line with the PROCESS criteria [5].

**Table 1:** Demographic and clinical characteristics of pediatric fibromatosis patients.

Factors	n(%)
**Age (years)**	
Median (IQR)	6 (5-11)
**Gender**	
Male	6 (85.7)
Female	1 (14.3)
**Site of tumor**	
Gluteal region	4 (57.1)
Distal forearm	1 (14.3)
Axilla	1 (14.3)
knee	1 (14.3)
**Margin status**	
Positive	4 (57.1)
Negative	3(42.9)
**Recurrence**	
Yes	4 (57.1)
No	3(42.9)

## 3 Discussion

Our study replicated the findings of several other studies regarding AF in the pediatric population. The median age of the participants of study was 6 years which was consistent with the findings from other studies that indicates that fibromatosis can affect any age group but it is more evident among children. However, it is reported that about 25% of all AFs occur among children of less than 15 years of age [6]. The possible explanation of this can be that the histo-morphology and biological behavior of AFs among children is more aggressive as compared to adults due to the high cellularity of the tumor [6].

This study results also indicate that majority of the children who had aggressive fibromatosis (AF) were boys. Literature also suggest that AF is higher among males [7]. Although there is dearth of information regarding the biology of AF, but literature suggests that targeting endocrine-mediated proliferation and other signaling pathways has yielded development of treatments with some clinical efficacy. It is also observed that anti-estrogen therapy exhibits anti-proliferative activity in such tumors. Hormonal therapies such as tamoxifen, toremifene, megestrol, progesterone, testolactone, and goserelin have shown improvement with the prognosis of such tumors [4]. Hence, the possible explanation of males been affected by this tumor as shown in our study and literature may be due to the involvement of some hormonal factors. However this needs further to exploration through large scale studies specially among pediatric population.

Majority of the patients in our study had tumor located in the gluteal region followed by the extremities. Literature also indicates that majority of these tumors occur at extremities followed by abdominal, thoracic and rarely in the head and neck [8].

Additionally, our study results also suggested that after surgery a high number of individuals had positive tumor margin and all of them had recurrence of disease. A study indicated a higher proportion of children who had positive margins after primary surgery developed disease recurrences versus those with negative margins [7]. Therefore, the high risk of disease recurrence among patients with positive margins indicates that there is a need of exploration of the role of the adjuvant treatment in such patients. A study conducted in Netherland on a sample of 13 children suggested that younger children (0–18 years) who received radiotherapy showed less locoregional control of disease however, when the dose was increased above 55 Gy, there was an improvement in the outcome but on the contrary the complications were increased. On the contrary in our country, the concept of adjuvant treatment is still ambiguous and post radiation therapy complications could be a reason. Moreover, studies also suggest that the recurrence rate in children varies from 23%–83% which results in multiple resections. Furthermore, studies also suggests that negative surgical margins causes decrease in the recurrence of AF [4,7,8].

The overall survival status of our study patients is 100% at 15 years, there is no death reported for the patients who were on follow up and this survival pattern is comparable to the survival status reported in literature [8]. All of the patients in our study were in regular follow up with us.

Patients in our study had recurrence and majority of the patients had not received any adjuvant therapy except one patient who had multiple recurrences. Literature suggests that adjuvant therapy must be considered for patients with residual disease and among those who have recurrent, progressive, or unresectable tumor. Traditional cytotoxic and non-cytotoxic agents are the chemotherapeutic therapy of choice [9]. However, there is still a need of multicenter, prospective (randomized) trials to clarify the role of adjuvant treatment for patients with pediatric AF [3].

## 4 Conclusions and recommendations

The conclusion of our study is that AF is commonly found among children below 15 years of age, affecting males more as compared to females. Positive margins after surgery indicate a high risk for disease recurrence therefore; primary surgery with negative margins is the treatment choice for children with AF. However, radiation may assist in maintaining local control of these tumors in patients with positive margins or residual disease therefore use of adjuvant therapy in children with AF may be a reasonable alternative. However, multicenter prospective (randomized) trials will be necessary to clarify the role of adjuvant treatment for patients with pediatric AF.

## Assistance with the study

None.

## Conflicts of interest

There are no potential conflicts of interest.

## Funding source

Not applicable.

## Ethical approval

This study was exempted by Aga Khan University Hospital ethics committee. ERC exemption no: 5066-Sur- ERC-17.

## Consent

Written informed consent was obtained from all of the patient’s parents/guardians, as all of the patients are minors, for publication of this case series and accompanying images. A copy of the written consents is available for review by the Editor-in-Chief of this journal on request.

## Author contribution

Dr Masood Umer: study concept and final review.

Javeria Saeed: study designing data collection, and writing.

Dr Nida Zahid: study interpretation and writing.

## Registration of research studies

UIN: Researchregistry4098.

## Guarantor

Masood Umer.

## Provenance and peer review

Not commissioned, externally peer-reviewed
